# Treatment outcome of NSCLC patients with *BRAF*^*non-V600E*^ mutations: a retrospective, multicentre analysis within the national Network Genomic Medicine (nNGM) Lung Cancer in Germany

**DOI:** 10.1016/j.esmoop.2025.105124

**Published:** 2025-07-14

**Authors:** C. Kropf-Sanchen, A. Rasokat, P. Christopoulos, C. Wenzel, T. Wehler, M. Rost, J. Kulhavy, N. Reinmuth, C. Schulz, M. Scheffler, J. Wolf, R. Büttner, S. Merkelbach-Bruse, M. Thomas, A. Stenzinger, M. Schütz, A. Bräuninger, M. Demes, H.-D. Hummel, N. Pfarr, N.T. Gaisa, J. Rawluk, E. Berezucki, K.T. Lutz, S. Galda, H. Jacobi, M. Collienne, M. Janning, T. Brummer, S. Loges, nNGM Lung Cancer

**Affiliations:** 1 Division of Pulmonology, Department of Internal Medicine II, Ulm University Medical Center, Ulm, Germany; 2 DKFZ-Hector Cancer Institute at the University Medical Center Mannheim, Mannheim, Germany; 3 Division of Personalized Medical Oncology (A420), German Cancer Research Center (DKFZ), German Center for Lung Research (DZL), Heidelberg, Germany; 4 Department of Personalized Oncology, University Hospital Mannheim, Medical Faculty Mannheim, University of Heidelberg, Mannheim, Germany; 5 Department for Internal Medicine, Center for Integrated Oncology Köln-Bonn, University Hospital Cologne, Cologne, Germany; 6 Department of Thoracic Oncology, Thoraxklinik, Heidelberg University Hospital and National Center for Tumor Diseases (NCT), NCT Heidelberg, a partnership between DKFZ and Heidelberg University Hospital, Heidelberg, Germany; 7 Translational Lung Research Center Heidelberg (TLRC-H), Member of the German Center for Lung Research, (DZL), Heidelberg, Germany; 8 Institute of Pathology, University Hospital Dresden, TU Dresden, Dresden, Germany; 9 Clinic for Internal Medicine, Justus-Liebig-University, Giessen, Germany; 10 Department of Medicine II, Hematology, Oncology, University Hospital Frankfurt, Frankfurt am Main, Germany; 11 Translational Oncology/Early Clinical Trial Unit (ECTU), Comprehensive Cancer Center Mainfranken and Bavarian Cancer Research Center (BZKF), University Hospital Würzburg, Würzburg, Germany; 12 Asklepios Lung Clinic, Member of the German Center for Lung Research (DZL), Munich-Gauting, Germany; 13 University Hospital Regensburg, Regensburg, Germany; 14 Institute of Pathology, Cologne University Hospital, Cologne, Germany; 15 Institute of Pathology, Heidelberg University Hospital, Heidelberg, Germany; 16 Clinic for Internal Medicine I and Pathology, University Hospital Dresden, TU Dresden, Dresden, Germany; 17 Institute of Pathology, Justus-Liebig-University, Giessen, Germany; 18 Molecular Pathology of the Dr. Senckenberg Institute for Pathology and Human Genetics & the University Hospital MVZ GmbH, Frankfurt, Germany; 19 Institute of General Pathology and Pathological Anatomy at the Technical University of Munich, Munich, Germany; 20 Institute of Pathology, University Hospital Ulm, Ulm, Germany; 21 Department of Internal Medicine I, Medical Center, Faculty of Medicine, University of Freiburg, Freiburg, Germany; 22 Institute of Molecular Medicine and Cell Research (IMMZ), Faculty of Medicine, University of Freiburg, Freiburg, Germany; 23 German Cancer Consortium (DKTK), Partner Site Freiburg and German Cancer Research Center (DKFZ), Heidelberg, Germany; 24 Comprehensive Cancer Center Freiburg (CCCF), Medical Center, Faculty of Medicine, University of Freiburg, Freiburg, Germany; 25 Center for Biological Signalling Studies BIOSS, University of Freiburg, Freiburg, Germany

**Keywords:** non-small-cell lung cancer, targeted therapy, *BRAF*^*non-V600E*^, MEK/ERK activation potential

## Abstract

**Background:**

Non-small-cell lung cancer patients with *BRAF*^*V600E*^ mutations benefit from targeted and (chemo-)immune therapy. However, treatment of BRAF^non-V600E^ mutations poses substantial challenges due to biological heterogeneity, different clinicogenomic features and limited therapy outcome data.

**Materials and methods:**

We conducted a retrospective analysis of *BRAF*^*non-V600E*^ mutation patients in the national Network Genomic Medicine Lung Cancer, assessing treatment outcomes upon targeted and (chemo-)immune therapy. Additionally, we evaluated mitogen-activated protein kinase (MEK)/extracellular-signal-regulated kinase (ERK) activation potential of selected, previously not characterized mutations *in vitro*.

**Results:**

Fifty-three patients with 11 different *BRAF*^*non-V600E*^ mutations undergoing targeted and/or (chemo-)immune therapy were identified. Patients with class I mutations achieved the longest progression-free survival (PFS) under targeted therapy [median PFS (mPFS) 9.8 months], whereas chemotherapy and chemoimmunotherapy led to an mPFS of 35 and 27 months, respectively. In patients with class II mutations, targeted therapy led to an mPFS of 6.3 months while chemotherapy, chemoimmunotherapy and immunotherapy resulted in an mPFS of 3.5, 3.7 and 3.6 months, respectively. Preclinical characterization demonstrated MEK phosphorylation potential and hence actionability of BRAF class II mutations G469A, G469R, G469V and *BRAF* K601. Patients exhibiting class III mutations did not respond to targeted therapy (mPFS 2.6 months), but showed responses to chemotherapy (mPFS 4.2 months), chemoimmunotherapy (mPFS 10.9 months) and immunotherapy (mPFS 7 months).

**Conclusions:**

Patients with activating BRAF^non-V600E^ mutations respond to BRAF/MEK inhibitor or (chemo-)immunotherapy, while those with non-activating mutations do not benefit from targeted therapy, but may benefit from (chemo-)immune therapy. Correlating preclinical activation assays with clinical outcomes can guide treatment decisions for patients with *BRAF*^*non-V600E*^ mutations, facilitating personalized treatment approaches.

## Highlights

• BRAF non-V600E mutations show diverse biological behavior in NSCLC.
• Class I/II BRAF mutations are more responsive to targeted therapies.
• Class III mutations needs alternative or combination treatment approaches.
• Molecular profiling is key to guide therapy in BRAFnon-V600E-mutant NSCLC.
• Personalized treatment improves outcomes in non-V600E BRAF NSCLC cases.

## Introduction

Non-small-cell lung cancer (NSCLC) is the leading cause of cancer-related mortality worldwide with adenocarcinoma representing the most frequent subtype.^1^ In this group in particular, the discovery of genetic alterations as oncogenic drivers has had a major impact on treatment options and patient prognosis over the last years.

Mutations in the *BRAF* gene, which occur in 2%-5% of NSCLC patients, have been identified as a therapeutic target for some time.^2^, ^3^, ^4^, ^5^ *BRAF* encodes for the protein serine/threonine protein kinase BRAF, which is involved in the regulation of the mitogen-activated protein kinase (MAPK)/extracellular signal-regulated kinase (ERK) signalling pathway and affects cell division and differentiation. The activating *BRAF*^*V600E*^ mutation occurs in 1%-3% of NSCLC cases and represents the most common *BRAF* alteration. For treating patients harbouring *BRAF*^*V600E*^ mutations, combinations of the BRAF inhibitor dabrafenib and the mitogen-activated protein kinase (MEK) inhibitor trametinib or encorafenib and binimetinib are approved as targeted therapies. These kinase inhibitors have shown substantial clinical benefit, with dabrafenib and trametinib inducing an objective response rate (ORR) of 63.9%, a median progression-free survival (mPFS) of 10.8 months and a median overall survival (mOS) of 17.3 months in treatment-naïve patients. For pretreated patients, the combined therapy led to an ORR of 68.4%, an mPFS of 10.2 months and an mOS of 18.2 months. The durable clinical benefit has been confirmed by recently published 4- and 5-year survival rates of 34% and 22% in treatment-naïve patients and of 26% and 19% in pretreated patients, respectively.^6^ For binimetinib and encorafenib, the PHAROS study showed an ORR of 75% with an mPFS of 30.2 months in treatment-naïve patients.^7^ For pretreated patients, an ORR of 46% was observed with an mPFS of 9.3 months^8^ and an mOS of 22.7 months.^7^ Other, recently presented data reported an ORR of 66.7% with an mPFS of 11.1 months for treatment-naïve patients in this setting.^9^

However, about half of the detected *BRAF* alterations account for *BRAF*^*non-V600E*^ mutations,^10^ which are increasingly identified due to the broad use of next-generation sequencing (NGS).^11^ This group is biologically very heterogeneous with different clinicogenomic features and not always fully understood in terms of BRAF^non-V600E^ properties as oncogenic drivers and direct druggability.^12^, ^13^, ^14^ There is currently no approval of any targeted therapy in this setting and experience is limited to individual case reports or data from small cohorts of patients, suggesting that selected patients may benefit from kinase inhibitor therapy.^15^, ^16^, ^17^ Preclinical studies suggest that classifying BRAF^non-V600E^ mutants by underlying kinase activity and RAF signalling mechanism could be helpful to predict treatment responses.^12^^,^^13^^,^^18^^,^^19^

Class I BRAF mutations affect amino acid residue V600 and signal in a RAS-independent fashion. They display high and constitutive BRAF kinase activity, even in their monomeric state, and are therefore highly transforming.^18^^,^^20^^,^^21^ Class II mutations contain elevated intrinsic kinase activity compared with wild-type BRAF and are considered to drive oncogenic transformation as RAS-independent dimers. Mutations generating class III BRAF oncoproteins impair or abolish intrinsic kinase activity. Nevertheless, these mutants efficiently dimerize with and activate catalytically competent RAF proteins, e.g. RAF1, thereby leading to paradoxical MEK/ERK signalling.^22^ This leads to a RAS dependence, and as a result, most tumours with class III mutations harbour alterations in *RAS* genes, their activators such as receptor tyrosine kinases (RTKs) or negative regulators, such as *NF1.*^13^^,^^22^^,^^23^ Similarly, BRAF^V600E^-selective inhibitors with a binding preference for RAF proteins in their monomeric state, including dabrafenib, can also cause paradoxical MEK/ERK pathway activation in the context of active RAS.^20^^,^^24^ Based on their binding mode, these compounds are also referred to as type I^1/2^ inhibitors.^25^ These insights identified novel indirect strategies, such as targeted therapy targeting receptor kinases upstream of RAS^9^ or the pan-RAF inhibitor sorafenib, either singly or in combination with trametinib, which prevents activation of the catalytically competent dimerization partner by kinase-dead (KD) BRAF.^26^ More recently, the detailed understanding into RAF inhibitor pharmacology induced a shift in drug development from the highly selective and monomer-restricted BRAF^V600E^ inhibitors vemurafenib, encorafenib and dabrafenib towards type II inhibitors targeting BRAF homo- and heterodimers such as naporafenib.^10^^,^^25^ Despite these insights, only class I mutants, in particular BRAF^V600E^, are well characterized in their response towards BRAF and MEK inhibitors, while very little is known about the *in vitro* responsiveness of BRAF class II or III mutations towards these compounds. Importantly, clinical data are very limited and almost exclusively restricted to BRAF^V600E^-driven NSCLC. In order to fill this gap, we assembled the thus-far largest cohort of patients with *BRAF*^*non-V600E*^ mutations in the national Network Genomic Medicine Lung Cancer (nNGM) in Germany and analysed their clinical outcome upon targeted therapy, chemotherapy and/or immune checkpoint inhibition. Furthermore, we evaluated the MEK/ERK activation potential of selected, previously not characterized mutations *in vitro*.

## Materials and methods

### Study population and data collection

In this retrospective, multicentre study, outcomes of patients with *BRAF*^*non-V600E*^ mutations upon different therapies including targeted therapy, chemotherapy, chemoimmunotherapy and immunotherapy were retrospectively analysed in the nNGM Lung Cancer in Germany. The aim of the multisectoral nNGM is to improve diagnostics and treatment for lung cancer patients, conducting quality-assured NGS panel diagnostics, therapy recommendations and clinical trials in >20 000 newly diagnosed NSCLC patients with advanced disease per year.^27^ Inclusion criteria for our study consisted of patients with non-resectable NSCLC (stage IIIB/IV) and presence of BRAF^non-V600E^ mutations eligible for systemic therapy.

*BRAF* mutations were classified into three groups based on current literature.^13^^,^^14^ Clinicopathological and outcome data were retrospectively collected, including patient characteristics, co-occurring gene mutations, type of systemic treatment (targeted therapy, chemo(immune)therapy or immune checkpoint inhibition), PFS and OS. Treatment response was assessed locally. For comparison, all treatment lines in our cohort were evaluated, focusing on the first treatment exposure of each regimen. Sample preparation and NGS were carried out locally according to the guidelines within the nNGM. The study was carried out in accordance with the Code of Ethics of the World Medical Association (Declaration of Helsinki). It received approval from the Ethics Committee II of the University of Heidelberg, Faculty of Medicine, Mannheim (no. 2023-837). Informed consent was obtained when required by law.

### Functional analysis

Class I variants are well documented as highly active^14^; consequently, no preclinical characterization was needed. We characterized class II non-V600E oncoproteins p.G469A, p.G469V and p.G469R regarding their sensitivity to dabrafenib and trametinib, further investigating the previously not yet sufficient preclinical data in these mutations. Due to a good response in two patients with *BRAF* K601E-mutant NSCLC, naporafenib was included in this analysis. We used HEK293T cells, a well-defined model system to interrogate the potential direct druggability of BRAF proteins within the same genetic background and without the interference by cell line-specific co-mutations or differentiation states.^28^ Further characterization of class III mutations was unnecessary due to sufficient existing preclinical data.^16^^,^^17^ Experimental procedures are described in the Supplementary Material, available at https://doi.org/10.1016/j.esmoop.2025.105124.

### Statistical analysis

PFS was calculated from the day of initiation of the respective systemic therapy until disease progression or death. OS was calculated from the day of first diagnosis to death. We analysed PFS and OS as main outcome parameters because ORRs were only available for a minority of patients. A response was considered as clinically meaningful, if the patient achieved a PFS of >3 months. Statistical analyses were carried out using SPSS version 29.0.2.0(20) (IBM Corp., New York, NY).

## Results

### Study design and participants

Ten nNGM centres (Cologne, Dresden, Frankfurt, Giessen, Heidelberg, Mannheim, Munich, Regensburg, Ulm, Würzburg) contributed 53 NSCLC cases in total, revealing 11 different *BRAF*^*non-V600E*^ mutations. Patients were treated between 2017 and 2023, 20 of them with targeted therapy. The median age was 66 ± 9.6 years; 24 patients (45%) were female, and 29 (55%) were male. Among them, 22 (41%) were current smokers (person who smokes cigarettes, cigars, pipes, or uses other tobacco or nicotine-containing products), 28 (53%) were former smokers (both with a median history of 40 pack-years) and 3 (6%) were never smokers (person who has never smoked or smoked fewer than 100 cigarettes in their lifetime). Nine patients (17%) had stage III disease not amenable to local treatment, while 44 (83%) had stage IV disease. Common metastatic sites included the lung (23%), brain (21%), bone (17%), liver and adrenals (15% each, as shown in Supplementary Figure S1; see Supplementary Table S1, available at https://doi.org/10.1016/j.esmoop.2025.105124).

### Classification of mutations

**Figure 1:**
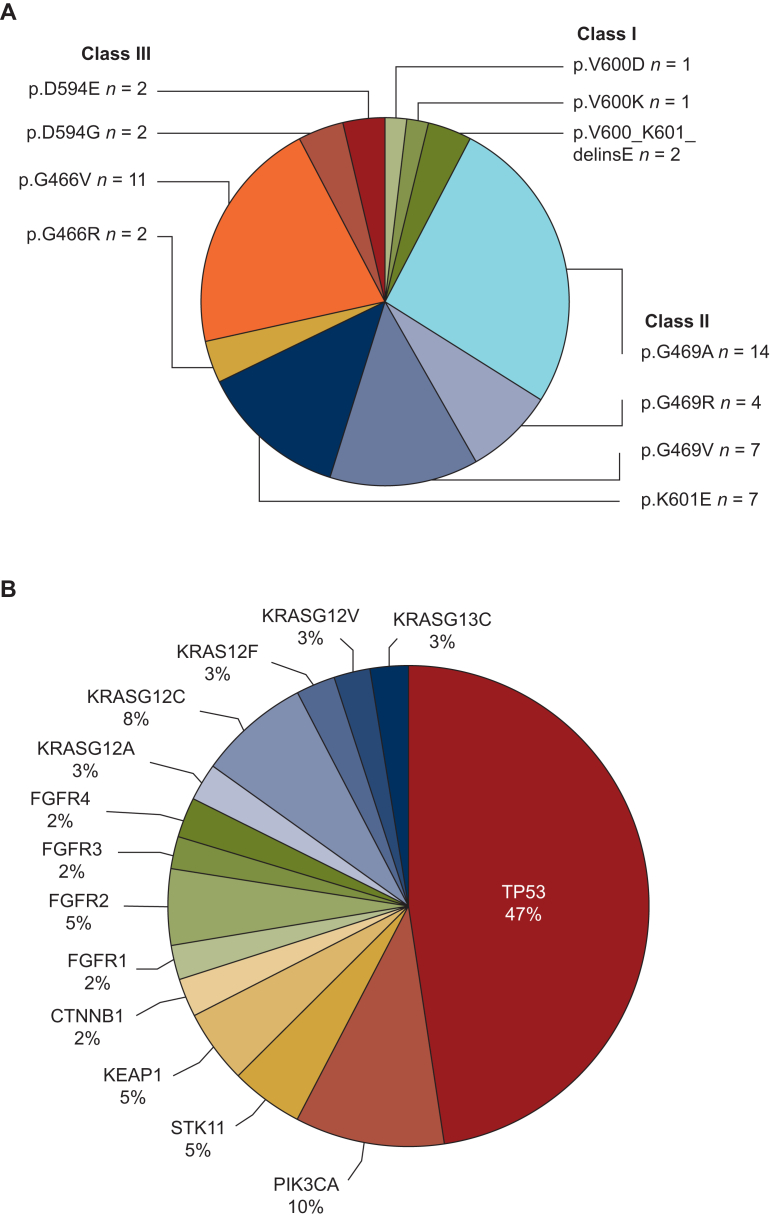
**Distribution of BRAF***^**non-V600E**^***-mutations, results of preclinical assays and therapeutic outcome in BRAF***^**non-V600E**^***-mutations.** Frequency and distribution of *BRAF*^*non-V600E*^ mutations (A) and co-mutations (B).

**Histology: *BRAF*^*non-V600E*^ mutations and co-mutations.** Detected class I BRAF^non-V600E^ mutations consisted of p.V600D and p.V600K (each *n* = 1) and p.V600_K601delinsE (*n* = 2). Among class II mutations, we found p.G469A (*n* = 14), p.G469R (*n* = 4), p.G469V (*n* = 7) and p.K601E (*n* = 7). Identified class III mutations comprised p.D594E (*n* = 2), p.D594G (*n* = 2), p.G466V (*n* = 11) and p.G466R (*n* = 2) (Figure 1A). Co-mutations in the *TP53* gene were present in 19 patients (36%); further co-mutations included *STK11, KEAP1, PIK3CA, CTNNB1* and different *KRAS* and *FGFR* mutations (Figure 1B).

### Outcome of the functional analysis

We tested the three non-V600E oncoproteins p.G469A, p.G469V and p.G469R for their dabrafenib and trametinib sensitivity in HEK293T cells. In the absence of inhibitors, BRAF^G469A^ displayed the highest MEK/ERK phosphorylation potential followed by G469V and G469R.

**Figure 2:**
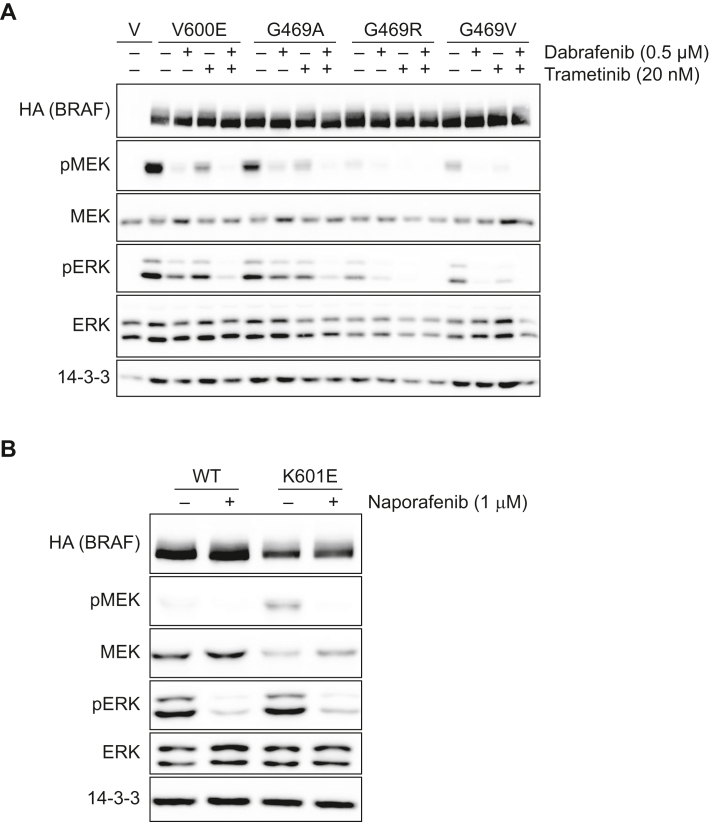
**Analysis of BRAF non-V600E Mutations: Effects on MEK Phosphorylation and Clinical Outcomes MEK phosphorylation potential of various non-V600E BRAF mutations (G469A, G469R, G469V, and K601E) and their modulation by targeted inhibitors**. Western blot showing the MEK phosphorylation potential of (A) BRAF G469A, G469R and G469V and its reduction by dabrafenib or trametinib and of (B) BRAF K601E with naporafenib. ERK, extracellular-signal-regulated kinase; HA, hemagglutinin; MEK, mitogen-activated protein kinase; WT, wild type.

The combination of dabrafenib 0.5 μM with trametinib 20 nM, a concentration ranging between reported peak and trough plasma levels,^29^^,^^30^ suppressed not only the MEK activation potential of BRAF^V600E^, but also that of all three other tested non-V600E oncoproteins. Our analyses also show that G469x mutations slightly differ in their signalling potential (Figure 2A). Furthermore, naporafenib suppressed MEK phosphorylation induced by p.K601E (Figure 2B).

### Clinical outcome under different therapeutic regimens

**Figure 3:**
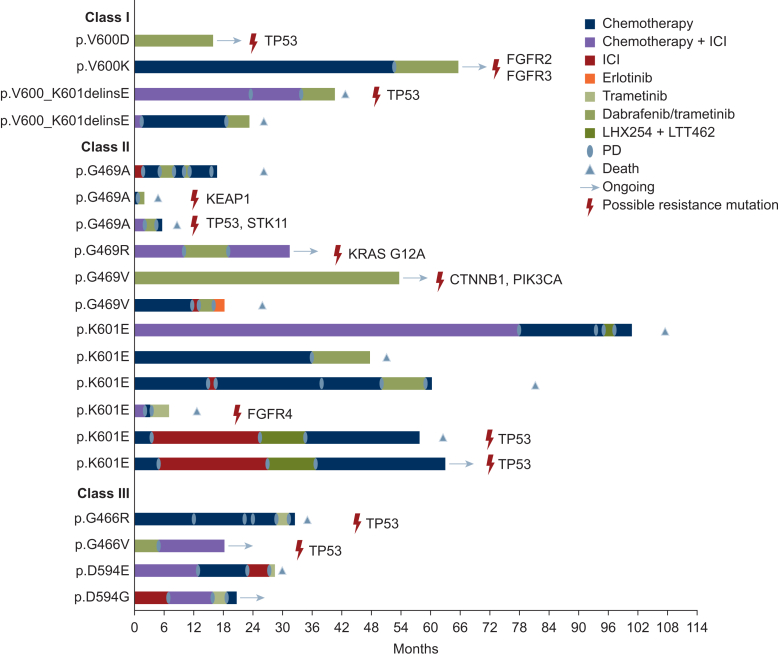
***BRAF*^*non-V600E*^ mutations, therapy lines and duration of response for patients receiving targeted therapy including possible resistance mutations.** ICI, immune checkpoint inhibition; LHX254, naporafenib; LTT462, rineterkib; PD, progressive disease.

In our cohort, 20 patients received targeted therapy, 14 with a combination of dabrafenib/trametinib, 4 with trametinib alone and 2 with the BRAF/RAF1 inhibitor naporafenib (also known as LXH254) in combination with the ERK1/2 inhibitor rineterkib (also known as LTT462) in a study setting. Targeted therapy was mostly used in later lines, with 25% in second-line and 40% in third-line therapy (Figure 3). Three patients had first-line targeted treatment (Figure 3). All four patients on dabrafenib monotherapy had progressive disease. The mPFS for the entire targeted therapy cohort was 4.6 months, ranging from 0.9 to 54 months. Overall, 34 patients received chemotherapy with an mPFS of 4.2 months, 23 received chemoimmunotherapy with an mPFS of 4.9 months and 20 received immunotherapy with an mPFS of 4.9 months.

#### Clinical outcome of patients with class I mutations

The four patients in this group being treated with dabrafenib and trametinib achieved an mPFS of 9.8 months under targeted therapy, ranging from 4.4 to an ongoing 15.6 months. Notably, two patients achieved partial response (50%) and two stable disease (50%). Chemotherapy (*n* = 2) and chemoimmunotherapy (*n* = 2) led to an mPFS of 35 months and 27 months, respectively.

#### Clinical outcome of patients with class II mutations

**Figure 4:**
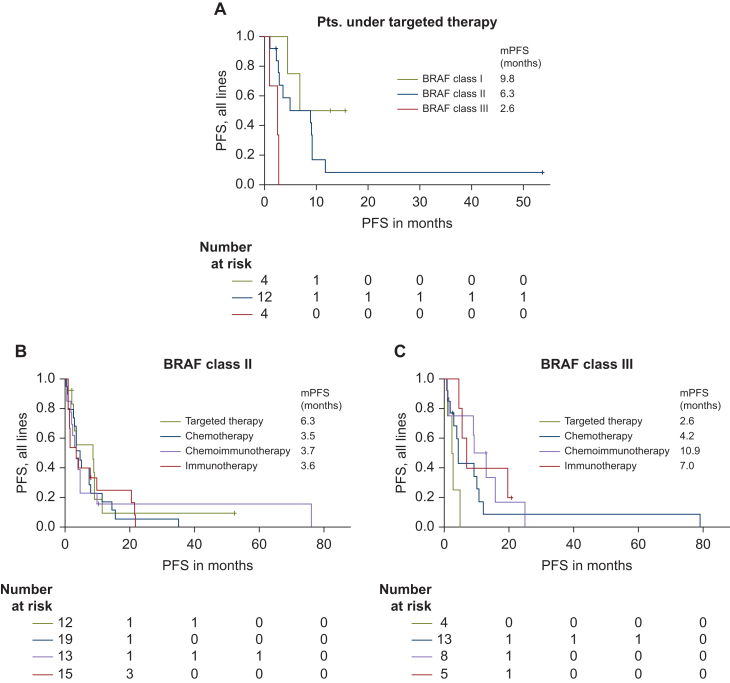
**Analysis of BRAF non-V600E mutations: effects on MEK phosphorylation and clinical outcomesprogression-free survival (PFS) under targeted therapies, stratified by BRAF mutation class and treatment regimen.** PFS under targeted therapy for different *BRAF* mutational classes in *non-V600E* mutations (A) as well as PFS under different therapeutic regimens for *BRAF* class II mutations (B) and BRAF class III mutations (C). mPFS, median progression-free survival; PFS, progression-free survival.

Thirty-two patients were included in this group, 12 receiving targeted therapy, resulting in an mPFS of 6.3 months (range 1-53.6 months). Notably, two patients treated with naporafenib and rineterkib showed durable responses with a PFS of 9.2 and 10 months. Chemotherapy, chemoimmunotherapy and immunotherapy resulted in an mPFS of 3.5, 3.7 and 3.6 months, respectively (Figure 4B).

#### Clinical outcome of patients with class III mutations

Expectedly, patients with class III mutations did not respond to targeted therapy (*n* = 4), indicated by an mPFS of 2.6 months. Under chemotherapy (*n* = 13), patients showed an mPFS of 4.2 months, whereas chemoimmunotherapy (*n* = 8) led to an mPFS of 10.9 months. For patients treated with mono-immunotherapy (*n* = 5), an mPFS of 7 months could be achieved (Figure 4C).

## Discussion

BRAF^non-V600E^ mutants exhibit biological heterogeneity, leading to diverse clinicogenomic features. Variances in kinase activity and RAF signalling mechanisms significantly impact the response to targeted therapy, including BRAF, RAF and/or MEK inhibition. While the nNGM includes a large number of NSCLC patients, only 20 with *BRAF*^*non-V600E*^ mutations receiving targeted therapy were identified. This finding reflects the rarity of these mutations and the infrequent use of targeted therapy as a therapeutic (off-label) option, also in part caused by the lack of information on the direct druggability of non-V600 BRAF mutants. However, our cohort, accompanied by functional characterization and a control group, represents the largest known group of *BRAF*^*non-V600E*^ NSCLC patients treated with targeted therapy. Our findings align with previous data, indicating highly variable responses to targeted therapy, with class I mutations benefitting most and class III mutations not showing any benefit (Figure 4A). All patients with class I and class II mutations who exhibited a prolonged response had received targeted therapy as first-line treatment. Currently, even for *BRAF^V600E^*, the data remain inconclusive: while some authors including Riely et al. and Planchard et al.^7^^,^^9^ have reported improved responses when targeted therapy is administered in the first line, others, like Wiesweg et al., have found no significant difference in efficacy between first-line treatment and its use in subsequent lines of therapy.^31^

### Class I mutations

Class I mutations are considered highly active^18^ and responses to treatment with vemurafenib were described previously.^32^ Due to a similar binding mode,^33^ a response to other type I^1/2^ inhibitors like dabrafenib is also expected. This was confirmed in our cohort with four patients achieving an mPFS of 9.8 months under targeted therapy, highlighting this regimen as a possible treatment option for patients with class I or structurally similar and highly active mutants. Patients also had a substantial clinical benefit from chemotherapy (mPFS 35 months) and chemoimmunotherapy (mPFS 27 months). Although numbers in our cohort are very low, our data are in line with previous publications showing that patients with class I mutations have longer PFS upon first-line chemotherapy than those with class II and III mutations.^34^ OS also proved to be longer for patients with classical *BRAF*^*V600E*^ mutations as compared with class II and III mutations, independent of targeted therapy.^34^ Despite the small numbers in our cohort, chemoimmunotherapy remains an approved and viable first-line option for patients with class I *BRAF* mutations.

### Class II mutations

Patients with class II mutations also showed a meaningful response to targeted therapy with an mPFS of 6.6 months. Unexpectedly, all three patients from our cohort with BRAF p.G469A did not respond to targeted therapy with an mPFS of only 2.0 months. BRAF^G469A^ is considered highly active^20^ and although there are a few case series not showing any response to vemurafenib,^32^^,^^35^ responses to dabrafenib and trametinib have been reported.^14^^,^^16^ In addition, our preclinical characterization of this mutation indicated suppression of MEK activation for dabrafenib and trametinib (Figure 2A). Responses in our patients could have been blunted by co-mutations—for example, one tumour harboured a *STK11* and a *TP53* mutation and another a *KEAP1* mutation. Furthermore, RTK alterations, *RAS* mutations or *NF1* deficiency could have been present and promoted paradoxical activity of dabrafenib in collaboration with activated RAS or could have relieved ERK pathway addiction by up-regulating alternative pathways.^22^^,^^36^^,^^37^ However, these data are unavailable to us as *NF1* mutations are not included in the NGS panel utilized in the nNGM.

For p.G469R, responses with sorafenib and dabrafenib/trametinib have been described.^15^^,^^17^ In our cohort, we observed a response in a patient harbouring this mutation, thus we can underscore the potential of targeted therapy in this particular situation. This is also true for p.G469V where we observed a remarkable outcome upon targeted therapy with dabrafenib and trametinib (PFS 53.6 months). Interestingly, vemurafenib alone could not induce clinical responses in this setting,^32^ which could be explained by its well-documented inability to inhibit non-V600E BRAF mutants due its specific effects on the drug accommodation influencing αC-helix.^25^^,^^38^ Dabrafenib, with its slightly different binding mode and higher affinity to RAF dimers than vemurafenib or encorafenib,^33^ on the other hand, has a much broader effect on various non-V600E oncoproteins, as is also reflected by preclinical studies and case reports.^14^^,^^28^^,^^39^ In the other BRAF^G469V^ case, which only benefitted from targeted therapy for 2.7 months, we detected a rare but activating *KRAS*^*G12F*^ mutation^40^ that might have caused paradoxical activation of the ERK pathway in the context of dabrafenib.

For p.K601E, reports in the literature concerning responses to targeted therapies are ambiguous with patients being unresponsive to vemurafenib.^32^ Under dabrafenib/trametinib, there have been reports of three cases, who achieved a partial remission, partly with a long lasting response with a PFS of 9 months.^41^, ^42^, ^43^ On the other hand, two cases were reported with a brief response of only 2 months or an immediate tumour progression.^44^^,^^45^ In our cohort, we found responses upon targeted therapy, especially under the investigational therapy with naporafenib and rineterkib. In our preclinical characterization, we were able to show suppression of BRAF^K601^-induced MEK signalling by naporafenib. Taking everything into consideration, targeted therapy represents an option as one line of treatment in patients with *BRAF*^*non-V600E*^ class II mutations, especially since treatment outcome upon chemotherapy, chemoimmunotherapy or immunotherapy was dismal in our cohort with an mPFS of 3.5, 3.7 and 3.6 months, respectively. We recommend chemo(immuno)therapy in this setting, recognizing that the supporting evidence is significantly less robust as compared with patients with class I mutations. Given the limited effectiveness of subsequent treatment options, we also advocate for a shared decision-making approach between clinician and patient—particularly for asymptomatic individuals—where observation may be considered as a viable option.

### Class III mutations

Patients with KD class III mutants were not responding to trametinib (mPFS 2.6 months) and had considerably better outcomes under chemoimmunotherapy and immunotherapy with an mPFS of 10.9 and 7 months, respectively. In retrospect, this could be explained by the pathomechanism of class III mutants. As these oncoproteins strictly require active RAS to induce paradoxical activation of their catalytically competent dimerization partner,^9^^,^^22^ it might be possible that the response of the two patients with D594x mutations and the case with the G466R substitution also contained either mutations in RAS or upstream activators such as RTKs, causing activation of other oncogenic pathways such as phosphoinositide 3-kinase (PI3K)/protein kinase B (AKT) or signal transducer and activator of transcription 3 (STAT3). As these pathways are not hit by trametinib, the poor response of these tumours could be potentially explained by co-mutations bypassing oncogene addiction to the RAF/MEK/ERK axis. Moreover, MEK inhibitors can even increase activity of the PI3K/AKT axis in preclinical models by various feedback mechanisms,^46^, ^47^, ^48^ suggesting that this compound might have further increased the activity of other oncogenic pathways. Unfortunately, however, this remains speculation as genetic data on these two D594x-mutant NSCLC other than the *BRAF* mutation status were not available. This highlights the necessity for more comprehensive genetic profiling of tumours, in particular those with class III *BRAF* mutations.

Nonetheless, we saw a response to targeted therapy in a patient with a p.G466V mutation with a PFS of 4.9 months. p.G466V is expected to paradoxically stimulate catalytically competent RAF proteins,^33^ leading to activation of the MEK/ERK axis, which is counteracted by trametinib. Retrospectively, this could explain the observed response in this patient and, as dabrafenib shows, compared with vemurafenib, a better efficacy against RAF dimers,^33^ it might be possible that this RAF inhibitor provided additional benefit as well. However, as dabrafenib is quite selective for BRAF^V600E^ and only inhibits wild-type RAF kinases at higher concentrations, we are not advocating here the use of dabrafenib to treat class III-driven tumours. In this case, we recommend a combination of a MEK and a pan-RAF inhibitor, e.g. sorafenib,^26^ or the investigational agent exarafenib or dual BRAF/RAF1 inhibitors like naporafenib or belvarafenib as they appear to be more promising based on their mode of action.^49^, ^50^, ^51^

### Proposal for a modified categorization of BRAF^*non-V600E*^ mutations in relation to a possible response to targeted therapy

In recent years, the categorization of *BRAF* mutations has proved to be very useful in understanding BRAF alterations in different cancer indications.^19^ To furnish clinicians with therapy recommendations, we put forward an adjustment based on our data, and notably, specialized preclinical characterization of all newly identified *BRAF*^*non-V600E*^ mutations.

**Figure 5:**
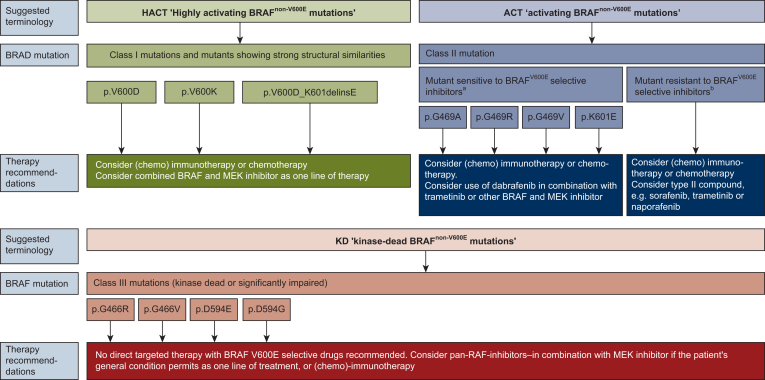
**Suggested modification of the classification of BRAF^non-V600E^ mutations based on their potential responsiveness to targeted therapy and therapeutic recommendation.**^a^Mutant is proven to be sensitive to BRAF^V600E^-selective inhibitors, e.g. dabrafenib, in preclinical characterisation and/or case reports or studies. ^b^Mutant is proven to be resistant to BRAF^V600E^-selective inhibitors, e.g. dabrafenib, in preclinical characterisation and/or case reports or studies.

We propose categorizing *BRAF*^*non-V600E*^ mutations based on their potential responsiveness to targeted therapy, labelling them as ‘highly activating (HACT) mutations’, ‘activating (ACT) mutations’ and ‘KD mutations’ (Figure 5). HACT mutations consist of class I mutations and mutants showing strong similarities, such as in our case p.V600_K601delinsE.^52^

Structure–function analyses identify BRAF^V600E^ as the prototypical class I mutant, characterized by RAS and dimerization independence.^10^ Mutations in its RAS binding or dimer interfaces have little effect on its high activity.^12^^,^^20^^,^^28^ Class I is a small group, mainly comprising V600E and in-frame InDels within the activation segment. For instance, p.V600_K601delinsE closely mimics V600E, as the inserted glutamate likely forms a similar salt bridge with K507, locking the kinase in its active conformation and disrupting inhibitory P-loop–activation segment interactions. In order to avoid confusion, we adhered to the nomenclature by Dankner et al.^19^ and would only consider V600E, V600D, V600K and V600_K601delinsE as highly similar to V600E. Therefore, they constitute the HACT group. For this group, combined targeted therapy with a BRAF inhibitor and a MEK inhibitor can be recommended as one line of treatment.

For ACT mutations, mainly class II, we propose a comprehensive characterization of each mutation, including well-defined cell culture experiments. Several of these mutants respond to dabrafenib, either singly or in combination with trametinib. Indeed, we have shown recently that even highly similar BRAF class II mutants generated by in-frame deletions of exon 12 are either dabrafenib sensitive or resistant.^28^ If a mutant is proven to be sensitive to a BRAF^V600E^-selective inhibitor in preclinical characterization and/or case reports or studies, we suggest considering the use of dabrafenib (rather than vemurafenib) in combination with trametinib or another BRAF and MEK inhibitor. Furthermore, type II compounds like naporafenib can be used to inhibit dabrafenib-resistant oncoproteins in cell culture models, including patient-derived organoids.^28^ In that regard, we have shown that naporafenib also inhibits BRAF^K601E^ in HEK293T cells and that this compound, together with the ERK inhibitor rineterkib, achieved durable responses in two of our patients with a PFS of 9.2 and 10 months. If a mutant is proven to be resistant to BRAF^V600E^-selective inhibitors, we recommend using a type II compound, e.g. sorafenib or naporafenib, or a MEK inhibitor like trametinib.

Thus, patients with class II mutants have the potential for a durable response to targeted therapy and therefore it should be part of a treatment concept in one line of therapy. This could become even more relevant as naporafenib, but also additional dimer-selective inhibitors, like belvarafenib, tovorafenib or exarafenib,^10^ are in advanced clinical testing and are more likely to inhibit class II mutants that are refractory to dabrafenib.

For the category of KD mutations, we would encompass all mutations that lack intrinsic kinase activity. Patients with tumours expressing these oncoproteins will not benefit from targeted therapy directed against BRAF, in particular if paradoxically acting type I^1/2^ compounds like dabrafenib are involved and the mutational status of RAS proteins or their activators or negative regulators such as NF1 is unknown.

Thus, targeted therapy with BRAF^V600E^-selective inhibitors should be avoided, especially as chemoimmunotherapy or immunotherapy offers very good other options. However, as also class III mutants trigger paradoxical MEK/ERK activation, trametinib or other MEK inhibitors represent a rationale, either singly or in combination with a pan-RAF inhibitor like sorafenib inhibiting their catalytically competent dimerization partner.^24^

This approach offers a more refined, treatment-oriented framework, as it integrates preclinical assessments with clinical data to guide therapeutic decisions more effectively. This linkage between molecular characterization and treatment outcomes represents a significant step forward in precision medicine.

We recognize various constraints in our analysis, such as its retrospective nature, the restricted number of cases and the clinical diversity within our cohort, coupled with the presence of a relatively small control group. Furthermore, *HRAS*, *NRAS*, *NF1*, *IRS*, *PTPN11/SHP2* and *YAP1* were not part of the nNGM NGS programme; therefore, this information has not been collected and we cannot comment on possible resistance mechanisms. Greater cohorts will be necessary to thoroughly assess the efficacy of targeted therapy in patients with *BRAF*^*non-V600E*^ mutations. Furthermore, since our data are based on a retrospective analysis of routine clinical practice, response assessments were not consistently carried out according to the current RECIST criteria. As a result, robust conclusions about response rates cannot be made.

Nonetheless, due to the rarity of the condition and the limited data available, our findings could still hold relevance for thoracic oncologists.

### Conclusion

Individuals harbouring HACT and ACT BRAF^non-V600E^ mutations exhibit effective responses to treatment with BRAF/MEK inhibitors and this treatment option should be considered as one line of treatment. Conversely, patients with KD mutations do not derive benefits from small molecule inhibitors developed for direct and selective BRAF inhibition, but rather show positive outcomes with (chemo-)immune therapy. Therefore, it is crucial to employ preclinical activation assays that demonstrate strong correlations with clinical outcomes before initiating targeted therapy in patients with unidentified or functionally and pharmacologically uncharacterized BRAF^non-V600E^ mutations as well as case discussions in molecular tumour boards. In summary, our study serves as a valuable guide for making treatment decisions and facilitating personalized treatment approaches.
