# Supplementary material for: Treatment outcome of NSCLC patients with BRAFnon-V600E mutations: a retrospective, multicentre analysis within the national Network Genomic Medicine (nNGM) Lung Cancer in Germany
Source: ESMO Open. 2025 Jul 14;10(8):105124. doi: 10.1016/j.esmoop.2025.105124 (PMC12281862; doi:10.1016/j.esmoop.2025.105124)
Supplement: Supplementary Data [file mmc1.pdf]

Tab.1: Patient Characteristics [SD: standard deviation, yrs: years;]

		**Patients (n=53) n [%]**
Median Age	[SD, yrs.]	66 +/- 9.6, [48-88 years]
Gender	female	24 [45]
	male	29 [55]
Smoking Status	current smoker	22 [41]
	ex-smoker	28 [53]
	never-smoker	3 [6], one patient each with mutation Class I (p.V600_K601delinsE), Class II (p.K601E) and Class III (p.G466V)
Clinical Stage	III	9 [17]
	IV	44 [83]
Site of metastasis	Lung, brain, bone, liver, adrenal	12 [23], 11 [21], 9 [17], 8 [15], 8 [15]
Histology	adenocarcinoma<br>not otherwise specified<br>squamous<br>large cell	46 [86]<br>3 [6]<br>2 [4]<br>2 [4]
Co-mutations		
BRAF Class I	*TP53*<br>*FGFR2, FGFR3*	2 [4]<br>each in 1 [2]
BRAF Class II	*TP53*<br>*STK11*<br>*PIK3CA, KEAP1, FGFR 4*<br>*KRAS G12A, G12C, G12F, G12V,*<br>*G13C*	8 [16]<br>2 [4]<br>each in 1 [2]<br>each in 1 [2]<br>1 [2]
BRAF Class III	*TP53*<br>*PIK3CA,*<br>*KRAS G12C*<br>*KEAP1,* CDKN2A, IDH1, FGFR1,<br>FGFR2,	9 [18]<br>2 [4]<br>2 [4]<br>each in 1 [2]<br>each in 1 [2]

Plasmids

The pMIG/HAhBRAF vectors encoding human hemagglutinin-tagged BRAFWT, *BRAF*^{V600E} and *BRAF* G469A were described previously[18]. The following pMIG/HAhBRAF plasmids were generated by standard *Pfu* ultra mediated site-directed mutagenesis with the following oligonucleotide pairs: G469R (5'-CTTTCCCTTGTAGACTGTTACAAATGATCCAGATCCAATTC-3'; 5'-CACTTTCCCTTGTAGACTGTTCGAAATGATCCAGATCCAATTC-3'), G469V (5'-GAATTGGATCTGGATCATTTGTAACAGTCTACAAGGGAAAG-3'; 5'-CTTTCCCTTGTAGACTGTTACAAATGATCCAGATCCAATTC-3') and K601E (5'-CACTTTCCCTTGTAGACTGTTCGAAATGATCCAGATCCAATTC-3'; 5'-CAAACTGATGGGACCCACTCCATCGAGATTCCACTGTAGCTAGACCAAAATCACC-3'). The correct HA-BRAF encoding cDNAs of all constructs were confirmed by sequencing.

Cell culture

HEK293T cells were provided by Andreas Hecht (IMMZ) and grown in DMEM medium (4.5 g/l glucose) supplemented with 10% fetal calf serum, 2 mM L-glutamine, 10 mM HEPES, 200 U/ml penicillin, 200 μg/ml streptomycin. All tissue culture media and additives were purchased from PAN Biotech (Aidenbach, Germany). Contamination with *Mycoplasma* was excluded by

Eurofins Genomics (Ebersberg, Germany). Kinase inhibitors were purchased from Selleck and dissolved in DMSO.

Western blot analyses

HEK293T cells were transfected as described previously[18]. Two days later, cells were treated with the indicated inhibitor concentrations for four hours prior to lysis in NP40 lysis buffer (50 mM Tris/HCl, pH 7.5; 1% NP40; 137 mM sodium chloride; 1% glycerol; 1 mM sodium orthovanadate; 0.5 mM EDTA; 0.01 mg/ml leupeptin, 0.1 mg/ml aprotinin, 1 mM AEBSF). Lysates were cleared by centrifugation (15.800 × g, 4 °C) for 10 min, mixed with 5 × Laemmli buffer (150 mM Tris pH 6.8; 20% glycerol; 15% sodium dodecylsulfate; 15% β-mercaptoethanol 0.01% bromophenol blue), boiled at 98 °C for 5 min prior to storage at -20 °C. Lysates were size-separated on 10% SDS-PAGE gels followed by tank blot transfer to Polyscreen polyvinyl difluoride membranes (Perkin Elmer). Proteins of interest were detected with the following primary antibodies: anti-phospho-MEK1/2 (#9121), anti-MEK1/2 (#9122), anti-phospho-ERK1/2 (#9101), anti-ERK1/2 (#9102); all from Cell Signaling Technology), anti-HA 3F10 (#11867431001) (Roche) and anti-pan-14–3–3 (#sc-1657; Santa Cruz Biotechnology). Bound primary antibodies were visualized using HRP-labelled secondary antibodies (Thermo Fisher) and a PeqlabTM Fusion Solo device and Fusion software (version 16.08).

Diagnostic Panels used in the nNGM network

Version 1: ALK (exon 22 - 25), BRAF (exon 11, 15) CTNNB1 (exon 3), EGFR (exon 18 – 21), FGFR1 (exon 4, 5, 6, 7, 10, 12, 13, 14,15), FGFR2 (Tr-A*: 6, 7, 8, 10, 11, 13, 14,15; Tr-B*: 8, 9, 12, 18), FGFR3 (exon 2, 5, 6, 8, 9, 11, 13, 15, 17) , FGFR4 (exon 3, 6, 9, 12, 13, 15, 16), HER2 (exon 2, 19, 20), IDH1 (exon 4 (R132X)), IDH2 (exon 4 (codon 140, 172)), KRAS (exon 2 – 4), MAP2K1 (exon 2, 3), MET (exon 16 - 19), NRAS (exon 2 – 4), PIK3CA (exon 10, 21), PTEN (exon 1-8), ROS1 (exon 34 – 41), TP53 (exon 4 – 8).

Version 2: ALK (exon 22 - 25), BRAF (exon 11, 15), CTNNB1 (exon 3), EGFR (exon 18 – 21), FGFR1 (exon 4, 5, 6, 7, 10, 12, 13, 14, 15), FGFR2 (Tr-A*: 6, 7, 8, 10, 11, 13, 14, 15; Tr-B*: 8, 9, 12, 18), FGFR3 (exon 3, 6, 7, 9, 10, 12, 14, 16, 18), FGFR4 (exon 3, 6, 9, 12, 13, 15, 16), HER2 (exon 8, 19, 20), HRAS (exon 2 – 4), IDH1 (exon 4 (R132X)), IDH2 (exon 4 (codon 140, 172)), KEAP1 (exon 2 – 6), KRAS (exon 2 – 4), MAP2K1 (exon 2, 3), MET (exon 14, 16 – 19 / Intron 13, first 100bp of intron 14), NRAS (exon 2 – 4), NTRK1 (exon 13 – 17), NTRK2 (exon 14 – 19), NTRK3 (exon 15 – 20), PIK3CA (exon 8, 10, 21), PTEN (exon 1-8), RET (exon 10 – 18), ROS1 (exon 34 – 41), STK11 (exon 1 – 9), TP53 (exon 4 – 8).

Version 3.2: ALK (exon 20 , 21, 22 – 28), BRAF (exon 11, 12, 14, 15), CTNNB1 (exon 3), CUL3, EGFR (exon 18 – 21), ERBB2 (exon 8, 19 – 21), FGFR1 (exon 4 – 7, 10, 12 – 16), FGFR2 (Tr-A*: 6, 7, 8, 10, 11, 13, 14, 15; Tr-B*: 8, 9, 12, 18), FGFR3 (exon 3, 6, 7, 9, 10, 12 – 16, 18), FGFR4 (exon 3, 6, 9, 12, 13, 15, 16), HRAS (exon 2 – 4), IDH1 (exon 4) , IDH2 (exon 4), KEAP1 (exon 2 – 6), KRAS (exon 2 – 4), MAP2K1 (exon 2, 3), , MET (exon 14, 16 – 19/ intron 13, first 100bp of intron 14), NFE2L2 (exon 1 –5), NRAS (exon 2 – 4), NTRK1 (exon 13 – 17), NTRK2 (exon 14 – 19), NTRK3 (exon 15 – 20), PIK3CA (exon 8, 10, 21), PTEN (exon 1-9), RB1 (all exons), RET (exon 10 – 18), ROS1 (exon 34 – 41), SMARCA4 (all exons), STK11 (exon 1 – 9), TP53 (exon 2 – 11).

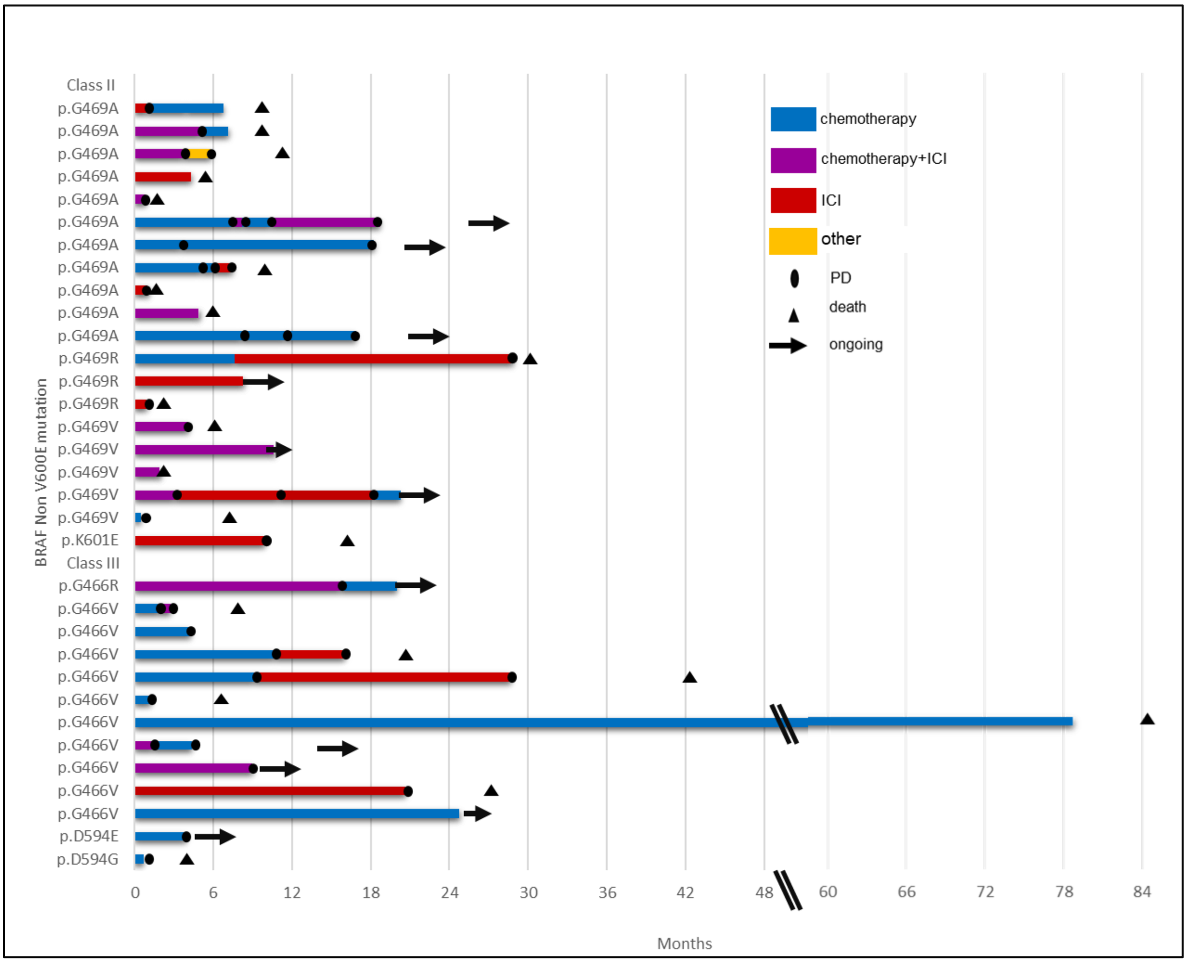

Fig. 1 ***BRAF*****$^{non\text{-}V600E}$ mutations, therapy lines and duration of response for patients not receiving targeted therapy** [ICI: immune checkpoint inhibition, PD progressive disease]
